# *N*,*N*-Dimethyl-3β-hydroxycholenamide Reduces Retinal Cholesterol via Partial Inhibition of Retinal Cholesterol Biosynthesis Rather Than its Liver X Receptor Transcriptional Activity

**DOI:** 10.3389/fphar.2018.00827

**Published:** 2018-07-25

**Authors:** Nicole El-Darzi, Artem Astafev, Natalia Mast, Aicha Saadane, Morrie Lam, Irina A. Pikuleva

**Affiliations:** Department of Ophthalmology and Visual Sciences, Case Western Reserve University, Cleveland, OH, United States

**Keywords:** *N*, *N*-dimethyl-3β-hydroxycholenamide, retina, cholesterol, Δ24-dehydrocholesterol, CYP27A1, CYP46A1, liver X receptor

## Abstract

*N*,*N*-dimethyl-3β-hydroxycholenamide (DMHCA) is an experimental pharmaceutical and a steroidal liver X receptor (LXR) agonist, which does not induce undesired hepatic lipogenesis. Herein, DMHCA was evaluated for its retinal effects on normal C57BL/6J and *Cyp27a1^−/−^Cyp46a1^−/−^* mice; the latter having higher retinal total and esterified cholesterol in addition to retinal vascular abnormalities. Different doses and two formulations were used for DMHCA delivery either via drinking water (C57BL/6J mice) or by oral gavage (*Cyp27a1^−/−^Cyp46a1^−/−^* mice). The duration of treatment was 1 week for C57BL/6J mice and 2 or 4 weeks for *Cyp27a1^−/−^Cyp46a1^−/−^* mice. In both genotypes, the higher DMHCA doses (37–80 mg/kg of body weight/day) neither increased serum triglycerides nor serum cholesterol but altered the levels of retinal sterols. Total retinal cholesterol was decreased in the DMHCA-treated mice, mainly due to a decrease in retinal unesterified cholesterol. In addition, retinal levels of cholesterol precursors lanosterol, zymosterol, desmosterol, and lathosterol were changed in *Cyp27a1^−/−^Cyp46a1^−/−^* mice. In both genotypes, DMHCA effect on retinal expression of the LXR target genes was only moderate and gender-specific. Collectively, the data obtained provide evidence for a decrease in retinal cholesterol as a result of DMHCA acting in the retina as an enzyme inhibitor of cholesterol biosynthesis rather than a LXR transcriptional activator. Specifically, DMHCA appears to partially inhibit the cholesterol biosynthetic enzyme Δ24-dehydrocholesterol reductase rather than upregulate the expression of LXR target genes involved in reverse cholesterol transport. The identified DMHCA dosages, formulations, and routes of delivery as well as the observed effects on the retina should be considered in future studies using DMHCA as a potential therapeutic for age-related macular degeneration and diabetic retinopathy.

## Introduction

**FIGURE 1:**
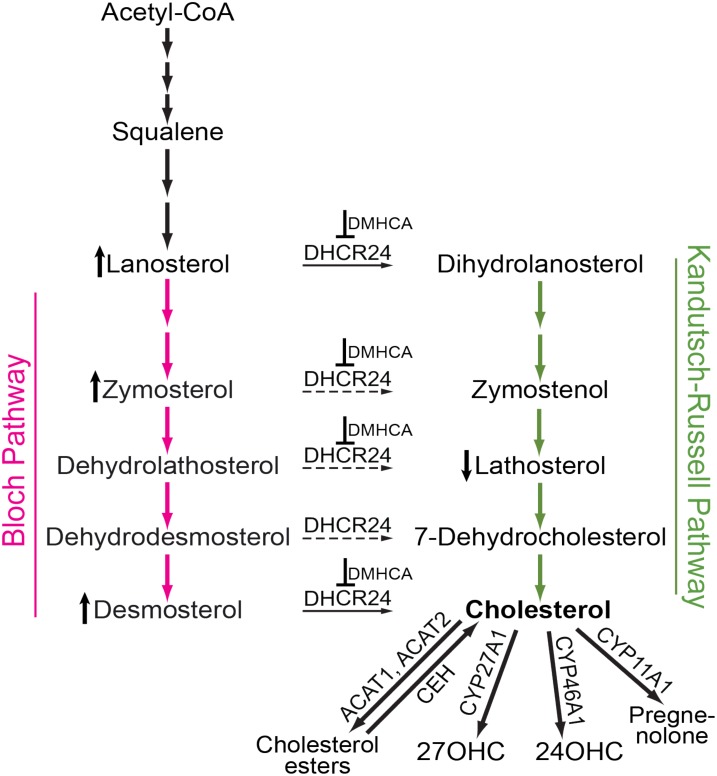
Schematic representation of cholesterol-related pathways of pertinence to the present work. Cholesterol biosynthesis starts from acetyl coenzyme A (Acetyl-CoA) and proceeds either via the Bloch (magenta arrows) or Kandutsch-Russell (green arrows) pathways. In certain tissues and cell types, there may be a switch from the Bloch to the Kandutsch-Russell pathway due to the action of Δ24-dehydrocholesterol reductase (DHCR24, solid and dashed arrows are the confirmed and potential points of the pathway crossover, respectively). Pharmacologic inhibition (for example with DMHCA, blunt-end arrows) may increase the levels of some of the DHCR24 substrates (e.g., lanosterol, zymosterol, and desmosterol, the vertical upward black arrow) and decrease the levels of some of the DHCR24 metabolites (e.g., lathosterol, the vertical downward black arrow), thus decreasing the levels of tissue cholesterol. If tissue cholesterol is in excess, it can be esterified by acyl-coenzyme A:cholesterol acyltransferases 1 and 2 (ACAT1 and ACAT2) and stored intracellularly in the form of lipid droplets. When cellular cholesterol levels are decreased, cholesterol esters are hydrolyzed to cholesterol by cholesterol ester hydrolases (CEH) with several enzymes being known to carry out this enzymatic reaction. Cholesterol excess can also be hydroxylated by CYP enzymes 27A1, 46A1, and 11A1 to yield 27-hydroxycholesterol (27OHC), 24-hydroxycholesterol (24OHC), and pregnenolone, respectively. These cholesterol derivatives either diffuse into the systemic circulation for delivery to the liver and subsequent degradation to bile acids or are metabolized locally at the site of production.

The retina is a thin tissue that lines the back of the eye and responds to light by converting it into an electrical signal. The retina is very small: only 1204 mm^2^ in humans and 15.6 mm^2^ in mice (Remtulla and Hallett, 1985; Panda-Jonas et al., 1994), a size that makes pharmacological investigations of the retina challenging. In addition, the retina is separated from the systemic circulation by the blood–retinal barrier, which may limit drug delivery to the retina. Lowering of cholesterol in the retina is one of the approaches that are currently under investigation for treatment of age-related macular degeneration (Pikuleva and Curcio, 2014), a common eye disease leading to vision loss in the elderly of industrialized countries. Indeed, significant amounts of cholesterol are present in drusen and subretinal drusenoid deposits (Curcio et al., 2001, 2005; Oak et al., 2014), the two hallmarks of AMD, and AMD risk factors include variants of several cholesterol-related genes (*ABCA1*, *APOE*, *CETP*, *LIPC*, *LPL*, *LRP6*, and *VLDLR*) (Miller, 2013). The majority (72%) of retinal cholesterol (at least in mice) is provided by local biosynthesis, which proceeds via the Bloch and Kandutsch-Russell pathways (**Figure 1**); the remaining 28% of cholesterol are taken up from the systemic circulation (Saadane et al., 2014; Lin et al., 2016). Retinal cholesterol elimination proceeds via metabolism to oxysterols catalyzed by CYP27A1 and CYP46A1 enzymes as well as lipoprotein-mediated reverse cholesterol transport to the liver (Tserentsoodol et al., 2006; Curcio et al., 2011; Fujihara et al., 2014; Pikuleva and Curcio, 2014). Additionally, small amounts of cholesterol excess stay in the retina in the form of cholesterol esters (Bretillon et al., 2008; Saadane et al., 2016).

*N*,*N*-dimethyl-3β-hydroxycholenamide (DMHCA) is an experimental pharmaceutical and a synthetic ligand for LXRs (Janowski et al., 1999; Spencer et al., 2001), a family of transcription factors. LXRs act as cellular sterol sensors and are activated by desmosterol (a cholesterol precursor) and certain oxysterols (metabolites of cholesterol) (Janowski et al., 1996; Chen et al., 2007; Spann et al., 2012). The two isoforms of this family, LXRα and LXRβ (Alberti et al., 2000), bind the same ligands and either increase (gene transactivation) or decrease (gene transrepression) the expression of the target genes (Glass and Ogawa, 2006; Kalaany and Mangelsdorf, 2006; Zelcer and Tontonoz, 2006). Originally, LXRs were discovered as nuclear receptors that limit cholesterol content by upregulating the expression of the genes involved in reverse cholesterol transport (e.g., *Abca1, Abcg1*, and *Apoe*). Later, LXR target genes were identified in the pathways of fatty acid biosynthesis (e.g., *Srebp1c, Acc1, Acc2, Fasn*, and *Scd1*), cholesterol uptake (*Idol*), glucose metabolism (*Glut4*), neovascularization (*Vegf*), and immune/inflammatory responses (e.g., *ArgII*, *Cox-2*, *iNos*, and *Il-6*) (Calkin and Tontonoz, 2012). In the latter, the proinflammatory genes (e.g., *Cox-2*, *iNos*, and *Il-6*) were downregulated by LXRs in contrast to the upregulation of cholesterol-, fatty acid-, and glucose-related genes (Glass and Ogawa, 2006; Zelcer and Tontonoz, 2006). Both gene transactivation and transrepression represent LXR activities that are of therapeutic importance (Hong and Tontonoz, 2014), with the exception of the induction of fatty acid synthesis in the liver, which is reflected by an increase in serum triglyceride levels. Significant efforts are directed toward developing LXR ligands that lack the undesired upregulation of hepatic lipogenesis and bind LXRs in a tissue-, pathway-, and isoform-specific manner (Hong and Tontonoz, 2014).

*N*,*N*-dimethyl-3β-hydroxycholenamide was initially synthesized for a study of structural requirements for the LXR ligands (Janowski et al., 1999). Later, this compound was found to activate the tested LXR target genes in the liver, small intestine, and peritoneal macrophages of C57BL/6 mice while having only a minor effect on hepatic fatty acid biosynthesis and serum triglyceride levels (Quinet et al., 2004). Subsequent DMHCA evaluation on *Apoe^−/−^* mice confirmed the desired upregulation of the LXR target genes and a lack of undesired serum hypertriglyceridemia; this evaluation also documented the antiatherogenic properties of DMHCA (Kratzer et al., 2009). A favorable pharmacologic profile of DMHCA was established and prompted further investigations of this compound (Caldas et al., 2011; Patel et al., 2014; Yu et al., 2016; Muller et al., 2017). Unexpectedly, in addition to LXR binding, DMHCA was discovered to inhibit DHCR24 both *in vitro* and *in vivo* (Pfeifer et al., 2011). DHCR24 catalyzes Δ^24^ reduction of the side chain of cholesterol precursors during cholesterol biosynthesis (**Figure 1**) and links the Bloch and Kandutsch-Russell pathways (Zerenturk et al., 2013; Mitsche et al., 2015). DMHCA inhibition of DHCR24 opened new pharmacologic options including those for treating hepatitis C viral infection, certain forms of cancers, and atherosclerosis (Muller et al., 2017). Herein, we evaluated DMHCA for its effect on the retina in mice. We found that under the treatment paradigms employed, DMHCA partially inhibited DHCR24 in the retina and reduced retinal cholesterol but did not seem to upregulate the expression of the LXR target genes.

## Materials and Methods

### Materials

DMHCA, lanosterol, [26,26,26,27,27,27-^2^H_6_]lanosterol, zymosterol, [2,2,3,4,4,-^2^H_5_]zymosterol, lathosterol, and desmosterol were from Avanti Polar Lipids, Inc. (Alabaster, AL, United States). Cholesterol and [^3^H]cholesterol were from Steraloids, Inc. (Newport, RI, United States) and PerkinElmer (Waltham, MA, United States), respectively. [26,26,26,27,27,27-^2^H_6_]Desmosterol and [1,2,5,6α-^2^H_4_]lathosterol were from C/D/N Isotopes Inc. (Pointe-Claire, QC, Canada). Rodent chow (5P76 Prolab Isopro RMH 3000) was from T. R. Last Co. (Saxonburg, PA, United States). All other chemicals were from Sigma-Aldrich (St. Louis, MO, United States) unless otherwise indicated. Recombinant CYP27A1, CYP46A1, adrenodoxin reductase, adrenodoxin, and NADPH-CYP oxidoreductase were expressed and purified as described (Sagara et al., 1992, 1993; Hanna et al., 1998; Mast et al., 2006, 2012).

### Animals

All animals were 4-month old. C57BL/6J mice were obtained from the Jackson Laboratory (Bar Harbor, ME, United States), and *Cyp27a1^−/−^Cyp46a1^−/−^* mice were generated as described (Saadane et al., 2014). Both genotypes were free of the *Crbl^rd8^* mutation. Mice were maintained on a standard 12-h light (approximately 10 lux)-dark cycle and were fed standard rodent chow and water *ad libitum*. Rodent chow contained only 0.02% cholesterol (w/w). All animal procedures were approved by the Case Western Reserve University Institutional Animal Care and Use Committee and have been carried out in accordance with the Guide for the Care and Use of Laboratory Animals as adopted and promulgated by the U.S. National Institutes of Health.

### Pharmacologic Treatments

All experiments were initiated in the morning. C57BL/6J mice received DMHCA in drinking water. *Cyp27a1^−/−^Cyp46a1^−/−^* mice received DMHCA by oral gavage. For delivery in drinking water, DMHCA stocks (84.3, 16.9, and 33.7 mg/l) were prepared for groups 1–3, respectively, in aqueous 11% 2-hydroxypropyl-β-cyclodextrin (HPCD) and then were diluted 10-fold with drinking water. This DMHCA-containing water was provided to mice for 7 days *ad libitum*. The control group received 1.1% HPCD in drinking water. On day 7, mice were fasted overnight but had access to water and were sacrificed the following morning. For delivery by gavage, DMHCA stock (100 mg/ml) in ethanol was prepared and diluted 10-fold with aqueous 5% HPCD. Animals received from 0.17 to 0.23 ml of 10 mg/ml DMHCA in aqueous solution of 10% ethanol and 4.5% HPCD so that the administered dose of DMHCA was equivalent to 80 mg/kg of BW. Animals underwent daily gavage for 15 or 29 days. The control group received aqueous solution of 10% ethanol and 4.5% HPCD. On day 14 or 28, mice were fasted overnight and sacrificed the next morning 1 h after the 15th or 29th gavage administration.

### Serum Analyses

Blood was withdrawn via cardiac puncture after mice were anesthetized via intraperitoneal injection of 80 mg/kg ketamine (Animal Health, Fort Dodge, IA, United States) mixed with 15 mg/kg xylazine (Akorn Inc., Lake Forest, IL, United States) in phosphate buffered saline, pH 7.4. The serum was isolated as described (Mast et al., 2010) and analyzed for total cholesterol and triglycerides by Marshfield Labs (Marshfield Clinic, Marshfield, WI, United States).

### Tissue Isolation and Sterol Quantifications

Mouse liver and retinas were isolated and processed as described (Zheng et al., 2015). Sterol quantifications were performed by isotope dilution gas chromatography-mass spectroscopy using deuterated sterol analogs as internal standards (Mast et al., 2011). Both unesterified and total retinal cholesterol (the sum of unesterified and esterified cholesterol) were measured.

### Quantitative Real Time PCR

Total RNA (1 μg) from the samples of individual livers or pooled retinas was isolated as described (Zheng et al., 2015) using the TRIzol Reagent (Life Technologies, Grand Island, NY, United States). This RNA was then converted to cDNA by SuperScript III reverse transcriptase (Invitrogen, Carlsbad, CA, United States) according to the manufacturer’s instructions. PCR reactions were performed in triplicate and were normalized to GAPDH. The primer sequences are shown in **Table 1**. Changes in relative mRNA level were calculated by the 2^−ΔΔCt^ method (Pfaffl, 2001).

**Table 1:** Primer sequences for quantitative real-time PCR.

Gene	Forward primer (5′–3′)	Reverse primer (5′–3′)
*Abca1*	AGGCCGCACCATTATTTTGTC	GGCAATTCTGTCCCCAAGGAT
*Abcg1*	ATTTCATCGTCCTGGGCATCT	CGGATTTTGTATCTGAGGACGAA
*Acc1*	TGTCCGCACTGACTGTAACCA	TGCTCCGCACAGATTCTTCA
*Acc2*	TCTTCCTGTCCGCCATCG	GGACGCCATACAGACAACCTTG
*Apoe*	GGCCCAGGAGAATCAATGAG	CCTGGCTGGATATGGATGTTG
*ArgII*	GACCACAGCCTGGCAATAGGT	TCAACCCAGATGACACAGAGATCT
*β-Actin*	TGTTACCAACTGGGACGACAT	TTGTAGAAGGTGTGGTGCCAGA
*Cox-2*	TGACCCCCAAGGCTCAAATA	CCCAGGTCCTCGCTTATGATC
*Fasn*	TCCTGGAACGAGAACACGATCT	AGAGACGTGTCACTCCTGGACTT
*Gapdh*	AGTCCATGCCATCACTGCCACC	CCAGTGAGCTTCCCGTTCAGC
*Idol*	GGAGCATGTCCAGCACGTCTA	GTGCAGGACGCATCAGATGA
*Il-6*	AGTTGCCTTCTTGGGACTGA	TCCACGATTTCCCAGAGAAC
*Inos*	GCCACCAACAATGGCAACA	CGTACCGGATGAGCTGTGAA
*Scd1*	CAACACCATGGCGTTCCA	AGGGTCGGCGTGTGTTTCT
*Srebp1c*	ACGGAGCCATGGATTGCA	AAGTCACTGTCTTGGTTGTTGATGA
*Vegf*	TGTGCAGGCTGCTGTAACGAT	CGCATGATCTGCATGGTGAT

### CYP27A1 and CYP46A1 Enzyme Assays

CYP27A1 and CYP46A1 were evaluated for cholesterol 27- and 24-hydroxylation, respectively. The conditions for each enzyme assay were as described (Mast et al., 2012; Lam et al., 2018). For each P450, the cholesterol concentration was equal to 0.5 *K*_m_ for cholesterol (2.3 and 2.7 μM for CYP27A1 and CYP46A1, respectively) (Mast et al., 2008, 2015), and DMHCA concentrations were equal to 45 and 43 μM for CYP27A1 and CYP46A1, respectively. Cholesterol was added from a 1 mM stock in 4.5% aqueous HPCD, and DMHCA was added from an 8 mM stock in 11% HPCD. Control incubations contained 0.06% HPCD. Product formation was linear with time and P450 concentration. More than 75% of inhibition of the P450 activity in this enzyme assay is indicative of a strong inhibitor (Mast et al., 2012; Lam et al., 2018).

### Statistics

The data represent either the mean ± SEM or the mean ± SD; the number of animals (*n*) is indicated in each figure or figure legend. Data were analyzed either by a two-tailed, unpaired Student’s *t*-test or by one-way ANOVA followed by Dunnett’s test for *post hoc* analysis using GraphPad Prism software (La Jolla, CA, United States). Statistical significance was defined as ^∗^*P* ≤ 0.05; ^∗∗^*P* ≤ 0.01; ^∗∗∗^*P* ≤ 0.001.

## Results

### Pilot Studies on C57BL/6J Mice

**FIGURE 2:**
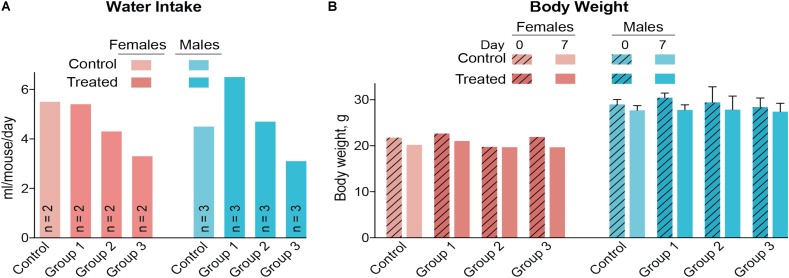
Water consumption **(A)** and body weight **(B)** of C57BL/6J mice treated with different DMHCA doses. The DMHCA doses are shown in **Table 2**. The duration of treatment was 7 days. DMHCA was delivered in drinking water containing 1.1% HPCD. The control group received drinking water containing 1.1% HPCD. The control and each dosing group was comprised of two female mice and three male mice with each gender housed in a separate cage. Water consumption was recorded at the end of treatment and divided by the number of mice per cage and duration of treatment. Results for the body weight measurements represent the mean for two female mice and the mean ± SD for three male mice.

A control and three treatment groups (1–3) were used, each comprised of two female and three male mice housed in two separate cages based on gender. The intended DMHCA doses were 20, 40, and 80 mg/kg of BW for groups 1–3, respectively, assuming that an average C57BL/6J mouse weighs ∼25 g and drinks ∼5.8 ml of water per day (Bachmanov et al., 2002). Thus, different amounts of DMHCA were added to drinking water for groups 1–3 (“Materials and Methods” section). However, the measurements of the intake of DMHCA-containing water (**Figure 2A**) revealed that both female and male mice had a trend toward a decreased water intake with increasing DMHCA concentrations; the lowest water intake being by group 3 (**Figure 2A**). In contrast, there did not seem to be a dependence of mouse BW on the administered DMHCA dose, except a slight decrease in BW at the end of the treatment in both female and male mice (≤10% and not statistically significant in male mice, **Figure 2B**). These data are in agreement with previous studies showing that DMHCA treatment may slightly decrease mouse BW (Kratzer et al., 2009; Patel et al., 2014). Thus, most of the actual DMHCA doses were lower than the intended doses (**Table 2**).

**Table 2:** DMHCA doses delivered in drinking water to C57BL/6J mice.

Gender	DMHCA dose, mg/kg BW/day		
	Group 1	Group 2	Group 3
Female mice	20	36	51
Male mice	18	27	37

*The control and each dosing group was comprised of two female mice and three male mice with each gender housed in a separate cage. Water consumption for each cage was recorded at the end of treatment and divided by the number of mice per cage and duration of treatment.*

**FIGURE 3:**
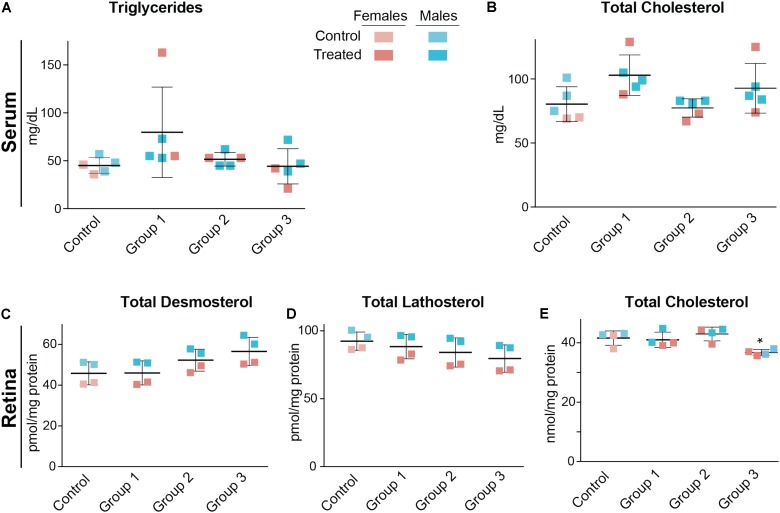
Serum **(A,B)** and retinal lipids **(C–E)** of C57BL/6J mice treated with different DMHCA doses. The DMHCA doses and treatment paradigm were the same as in **Figure 2**. The results are the mean ± SD of the measurements in the samples from individual animals of both genders. Asterisk is a significant change relative to the control group as assessed by one-way ANOVA followed by Dunnett’s test for *post hoc* analysis. ^∗^*P* ≤ 0.05.

Serum triglycerides and serum total cholesterol were measured in the vehicle-(control) and DMHCA-treated groups to confirm the lack of undesired liver lipogenesis (**Figures 3A,B**). No changes in the serum levels of these lipids were found, although we documented a higher interindividual variability in female mice than in male mice. Then, retinal cholesterol and retinal cholesterol precursors were quantified: desmosterol and lathosterol, which are sterol intermediates in the Bloch and Kandutsch-Russell pathways of cholesterol biosynthesis, respectively (**Figure 1**; Zerenturk et al., 2013). There was a trend toward an increased desmosterol levels and decreased lathosterol levels with increasing DMHCA doses (**Figures 3C,D**). Also, total cholesterol was decreased by 14% in group 3, which received the highest DMHCA dose (**Figure 3E**).

**FIGURE 4:**
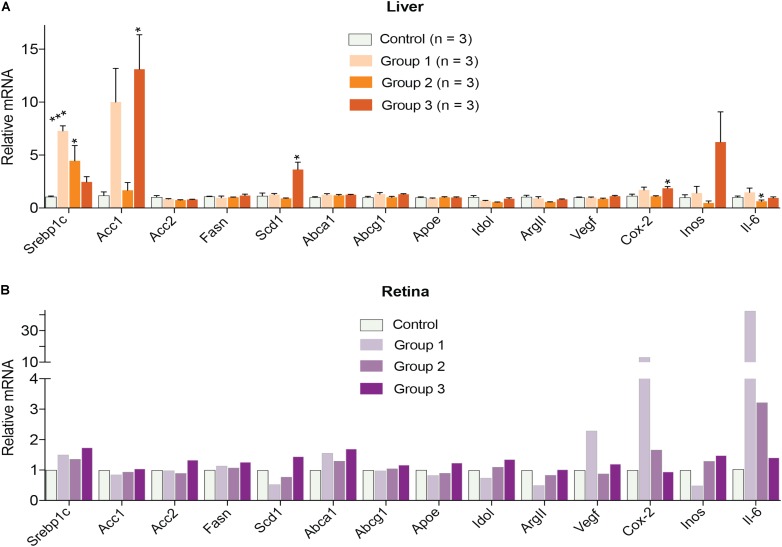
Hepatic **(A)** and retinal **(B)** gene expression in C57BL/6J mice treated with different DMHCA doses. The DMHCA doses and treatment paradigm were the same as in **Figure 2**. The results are the mean ± SEM of the duplicate measurements in the individual samples of the liver from two female one male mouse **(A)** or a pooled sample of five retinas from two female and three male mice **(B)**. Asterisks are significant changes relative to the control group as assessed by one-way ANOVA followed by Dunnett’s test for *post hoc* analysis. ^∗^*P* ≤ 0.05, ^∗∗^*P* ≤ 0.01, ^∗∗∗^*P* ≤ 0.001.

Finally, gene expression was evaluated in the liver and retina. Hepatic expression of LXR target genes were mostly unchanged, except a paradoxical dose-dependent decrease in the *Srebp1c* expression (**Figure 4A**). Additionally, *Acc1, Scd1*, and *Inos* in group 3 had the largest extent of upregulation. Yet, serum triglyceride levels were not increased in DMHCA-treated mice (**Figure 3A**) suggesting that increased *Srebp1c, Acc1*, and *Scd1* expression did not translate into increased liver lipogenesis. In the retina, a dose-dependent decrease seemed to occur only in the expression of pro-inflammatory genes *Cox-2* and *Il-6* with the remaining changes in gene expression did not seem to be dose-dependent (**Figure 4B**).

Thus, the results of the pilot dose-dependence studies were encouraging. They confirmed a lack of hypertriglyceridemia in mice treated with DMHCA doses of up to 51 mg/kg BW. They identified the effective DMHCA doses (37 and 51 mg/kg BW for male and female mice, respectively) that led to a decrease in total retinal cholesterol. Accordingly, we decided to test DMHCA on *Cyp27a1^−/−^Cyp46a1^−/−^* mice who develop retinal lesions similar to those found in type 3 neovascular age-related macular degeneration and early stage diabetic retinopathy (Saadane et al., 2014). Of importance was that the total retinal cholesterol in *Cyp27a1^−/−^Cyp46a1^−/−^* mice is twice as high as in wild-type mice on the same C57BL/6J;129S6/SvEv background (Saadane et al., 2014), and retinal cholesterol excess in this genotype is stored in the form of cholesterol esters (Saadane et al., 2016). Because of such high and unusual retinal cholesterol content, we decided to: (1) increase the DMHCA dose to 80 mg/kg BW/day; (2) increase the treatment time to either 2 or 4 weeks; and (3) switch from drug delivery in drinking water to gavage to prevent animal dehydration and ensure that every animal in its respective treatment group receives the same DMHCA dose.

### DMHCA Treatment of *Cyp27a1^−/−^Cyp46a1^−/−^* Mice

**FIGURE 5:**
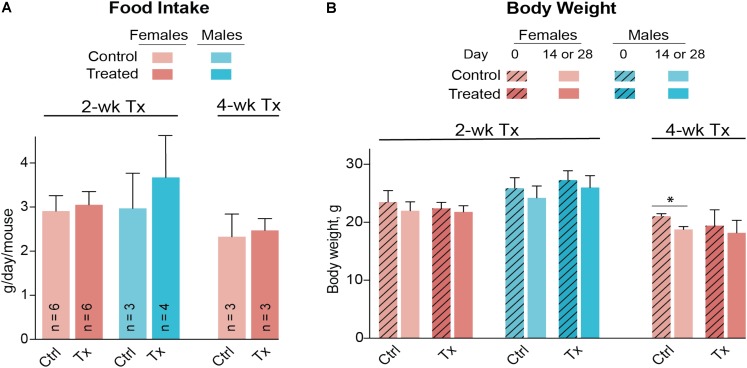
Food consumption **(A)** and body weight **(B)** of *Cyp27a1^−/−^Cyp46a1^−/−^* mice treated with DMHCA. DMHCA was formulated with aqueous solution of 10% ethanol and 4.5% HPCD and delivered by oral gavage at a 80 mg/kg BW/day dose for 2 or 4 weeks. The number of animals per group (n) is indicated in **A** and is the same in **B**. Each animal was housed in a separate cage; food consumption was recorded at the end of treatment and divided by duration of treatment. The results are the mean ± SD of the measurements in individual animals. Asterisks are significant changes relative to the control group as assessed by a two-tailed, unpaired Student’s *t*-test. ^∗^*P* ≤ 0.05. Ctrl, control; Tx, treatment.

Both female and male mice were used for the 2-week treatment study, and only female mice were used in the 4-week treatment experiment due to the female gender having a higher interindividual variability in their serum lipid profile (**Figures 3A,B**). DMHCA (80 mg/kg BW/day) was formulated with aqueous 10% ethanol/4.5% HPCD and delivered orally by gavage. Average food intake was the same in vehicle-treated (control) and DMHCA-treated mice (**Figure 5A**), and average BWs were not significantly different by the end of either 2- or 4-week treatment with the exception of the control group in the 4-week experiment that had a small decrease in the BW (**Figure 5B**).

**FIGURE 6:**
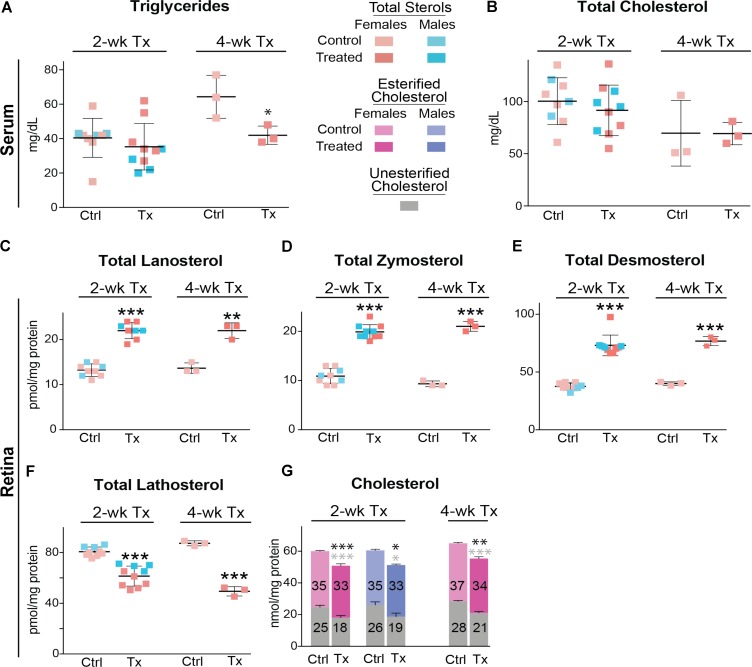
Serum **(A,B)** and retinal **(C–G)** lipids of *Cyp27a1^−/−^Cyp46a1^−/−^* mice treated with DMHCA. The treatment paradigm and number of animals per group were the same as in **Figure 5**. The results are the mean ± SD of the measurements in samples from individual animals of both genders. Numbers inside the bars in **G** represent the mean values for esterified and unesterified cholesterol; black and gray asterisks are significant changes relative to the control group for total and unesterified cholesterol, respectively, as assessed by a two-tailed, unpaired Student’s *t*-test. ^∗^*P* ≤ 0.05, ^∗∗^*P* ≤ 0.01, and ^∗∗∗^*P* ≤ 0.001. When female and male mice in a 2-week treatment were analyzed separately, statistical analyses did not reveal differences as compared to the group comprised of both genders. Ctrl, control; Tx, treatment.

Similar to C57BL/6J mice, *Cyp27a1^−/−^Cyp46a1^−/−^* mice did not show gender differences in the levels of serum triglycerides and total cholesterol (**Figures 6A,B**). Serum triglycerides were decreased by 35% after a 4-week treatment, whereas total serum cholesterol remained unchanged after 2 and 4 weeks of treatment. The most pronounced effect of DMHCA was on the levels of retinal sterols. The levels of total lanosterol, zymosterol, and desmosterol were increased by ≥70% in both the 2- and 4-week treatment groups as compared to the control groups (**Figures 6C–E**), whereas the lathosterol levels were decreased by up to 46% (**Figure 6F**). As compared to changes in retinal levels of cholesterol precursors, changes in the levels of total cholesterol in DMHCA-treated mice were smaller: only 15 and 17% in the 2- and 4-week treatment groups, respectively (**Figure 6G**). Remarkably, these decreases in total cholesterol mainly occurred due to a reduction in the levels of unesterified cholesterol with the levels of cholesterol esters being essentially unchanged.

**FIGURE 7:**
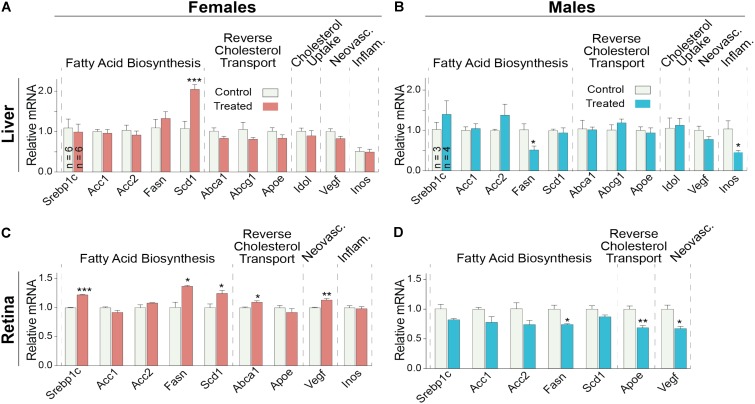
Hepatic **(A,B)** and retinal **(C,D)** gene expression in *Cyp27a1^−/−^Cyp46a1^−/−^* mice treated with DMHCA for 2 weeks. The treatment paradigm was the same as in **Figure 4**. **(A,B)** The results are the mean ± SEM of triplicate measurements in the individual samples of the liver from six female mice **(A)** and three to four male mice **(B)** or a pooled sample of individual retinas from six female mice **(C)** and three male mice **(D)**. Asterisks are significant changes relative to the control group as assessed by a two-tailed, unpaired Student’s *t*-test. ^∗^*P* ≤ 0.05, ^∗∗^*P* ≤ 0.01, ^∗∗∗^*P* ≤ 0.001.

Since the decrease in retinal cholesterol was similar in 2- and 4-week treated mice, the DMHCA effect on retinal gene expression was evaluated in 2-week treated mice. In the liver, DMHCA administration did not generally affect the expression of the studied genes (**Figures 7A,B**), except a 2-fold increase in the expression of *Scd1* in female mice and a ≥2-fold decrease in the expression of *Fasn* and *Inos* in male mice. In the retina, however, 5 out of the 9 studied genes in female mice (*Srepb1c*, *Fasn*, *Scd1*, *Abca1*, and *Vegf*) and 3 out of the 7 genes in male mice (*Fasn*, *Apoe*, and *Vegf*) showed an altered expression after DMHCA treatment (**Figures 7C,D**). Yet, these changes were moderate (up to 40%) and gender-specific: there was a pattern of increased gene expression in female mice, while in male mice, gene expression was decreased.

### DMHCA Effect on CYP27A1 and CYP46A1

CYP27A1 and CYP46A1 are the two major cholesterol hydroxylases in the retina that utilize cholesterol as a substrate to produce different metabolites, 27-hydroxycholesterol and 24-hydroxycholesterol (**Figure 1**), respectively (Saadane et al., 2014). The levels of retinal cholesterol were different in C57BL/6J and *Cyp27a1^−/−^Cyp46a1^−/−^* mice, and mice of the two genotypes received different DMHCA doses in different formulations. Nevertheless, DMHCA reduced retinal cholesterol in both genotypes by the same extent, 14 and 15–17%. Such an effect could be due to DMHCA inhibition of CYP27A1 and/or CYP46A1 in C57BL/6J mice, and thus a lack of cholesterol removal by metabolism in the retina of both genotypes. Hence, we used purified recombinant CYP27A1 and CYP46A1 and tested DMHCA for >75% enzyme inhibition in the *in vitro* enzyme assay, an indication of a strong P450 inhibitor (Lam et al., 2018). Neither CYP27A1 nor CYP46A1 were inhibited by DMHCA *in vitro* (**Table 3**), thus suggesting that retinal metabolism of cholesterol does not contribute to retinal cholesterol reduction as a result of DMHCA treatment.

**Table 3:** Effect of DMHCA on *in vitro* cholesterol hydroxylation by CYP27A1 and CYP46A1.

P450	P450 activity, %	
	no DMHCA	+ DMHCA
CYP27A1	100.0 ± 1.9	103.4 ± 3.8
CYP46A1	100.0 ± 5.4	109.9 ± 9.7

*The enzyme assays were carried out as described under the Section “Materials and Methods.” The results represent the mean ± SD of triplicate measurements.*

## Discussion

To the best of our knowledge, we have pioneered an *in vivo* study of retinal effects of DMHCA, an experimental steroidal LXR agonist. First, we conducted a pilot dose dependence study of DMHCA on a small group of mice from a common laboratory strain C57BL/6J (**Figures 2–4**). Then, we evaluated *Cyp27a1^−/−^Cyp46a1^−/−^* mice (**Figures 5–7**), which are on the C57BL/6J;129S6/SvEv background with total retinal cholesterol ∼1.5 times higher than that of wild-type mice on the C57BL/6J background. Retinal cholesterol excess in these animals is stored in the form of cholesterol esters, and their retinas have a number of pathologies (Saadane et al., 2014, 2016). Significant cholesterol esterification is unusual for the retina because normally, cholesterol esters are present in only very small amounts (up to 15%) both in mouse or human retina (Bretillon et al., 2008; Mast et al., 2011). Nevertheless, cholesterol esters are present in drusen, a hallmark of age-related macular degeneration, whereas subretinal drusenoid deposits, another hallmark of this disease, are mainly composed of unesterified cholesterol (Curcio et al., 2001, 2005; Oak et al., 2014). Hence, to be clinically relevant, evaluation of experimental pharmaceuticals for age-related macular degeneration should include the quantifications of different forms of retinal cholesterol: total, unesterified, and esterified cholesterol.

We found that at oral daily doses of 37 mg/kg BW given to male mice and 51 mg/kg BW given to female mice (**Table 2**), DMHCA decreased the levels of total retinal cholesterol in the C57BL/6J strain without affecting the levels of serum cholesterol (**Figures 3B,E**). Similarly, DMHCA (80 mg/kg BW/day) reduced retinal but not serum cholesterol in *Cyp27a1^−/−^Cyp46a1^−/−^* mice (**Figures 6B,G**). Collectively, these data suggest that DMHCA crosses the blood–retina barrier and reaches neural retina where it decreases cholesterol. Previously, DMHCA was shown to be readily absorbed in the intestine and distributed to different organs (Pfeifer et al., 2011). In C57BL/6 mice, the highest DMHCA concentration at an 80 mg/kg BW/day dose was found in the small intestine with lower drug concentrations in the kidney, heart, lung, and the liver (Pfeifer et al., 2011). In the brain, DMHCA was detected only in very small amounts and did not show the effects observed in the other organs (Pfeifer et al., 2011). Thus, unlike the blood–retina barrier, the blood–brain barrier seems to limit DMHCA availability to the brain. Conversely, in a mouse model of a traumatic brain injury, DMHCA (50 mg/kg BW) was reported to be well distributed in the brain and increase the expression of the LXR target *Abca1*, while decreasing the expression of pro-inflammatory *Il-1β* and *Tnfα* at the site of injury (Yu et al., 2016).

The retina is a neural tissue composed of both neurons and astrocytes. In mice, the main source of retinal cholesterol is local biosynthesis, which accounts for ∼72% of total retinal cholesterol (Lin et al., 2016). Retinal cholesterol biosynthesis proceeds via both, the Bloch and Kandutsch-Russell pathways because the metabolites from both pathways were detected in the retina in the same concentration range (Omarova et al., 2012). Desmosterol is a marker of cholesterol biosynthesis in astrocytes (Pfrieger and Ungerer, 2011) and is the last sterol intermediate in the Bloch pathway. Desmosterol is reduced by DHCR24 to yield cholesterol (**Figure 1**). The levels of this cholesterol precursor will increase, if DHCR24 is inhibited, because desmosterol will not be converted to cholesterol. Similarly, there may be an increase in the levels of lanosterol and zymosterol, the upstream desmosterol precursor and also the substrates for DHCR24. Lathosterol is a marker of cholesterol biosynthesis in neurons (Pfrieger and Ungerer, 2011) and is the penultimate sterol intermediate in the Kandutsch-Russell pathway (**Figure 1**). Lathosterol is a product of DHCR24 reduction of dehydrolathosterol, an intermediate in the Bloch pathway (Zerenturk et al., 2013; Mitsche et al., 2015). The levels of lathosterol will decrease, if DHCR24 is inhibited, because lathosterol will not be formed. Accordingly, an increase in retinal lanosterol, zymosterol, and desmosterol levels with a simultaneous decrease in retinal lathosterol levels (**Figures 6C–F**) suggest that treatment with an 80 mg/kg BW/day DMHCA dose partially inhibited DHCR24 and both pathways of retinal cholesterol biosynthesis. This interpretation is supported by previous studies on HepG2 cells treated with increasing DMHCA concentrations and supplemented with desmosterol as a substrate for DHCR24 (Pfeifer et al., 2011). Increasing DMHCA concentrations led to a decrease in cholesterol production by these HepG2 cells and an increase in desmosterol levels, thus providing evidence that DMHCA indeed inhibits DHCR24.

Retinal expression of genes involved in reverse cholesterol transport was either not increased or increased only moderately in DMHCA-treated mice (**Figures 7C,D**). Therefore, our sterol and mRNA quantifications collectively suggest that under the experimental conditions used, namely the DMHCA dose, formulation, route of delivery, and treatment time, this LXR agonist probably reduces retinal cholesterol (**Figure 6G**) via partial inhibition of DHCR24 rather than its transcriptional LXR activity controlling the expression of genes involved in reverse cholesterol transport. Moderate effects of DMHCA on retinal expression of LXR target genes (**Figures 7C,D**) are in agreement with experiments that directly compared the potency of DMHCA to T0901317 or GW3965, which are first generation non-steroidal LXR agonists that induce liver lipogenesis. When the same doses of DMHCA, T0901317, or GW3965 were used (50 or 80 mg/kg BW/day), T0901317 and GW3965 were more potent LXR agonists than DMHCA in the liver of C57BL/6 mice or in the C57BL/6 brain with traumatic injury (Quinet et al., 2004; Kratzer et al., 2009; Yu et al., 2016). We hypothesize that a higher, >100 mg/kg BW/day, DMHCA dose could possibly upregulate the reverse cholesterol transport genes in the retina upon oral DMHCA delivery.

Partial retinal DHCR24 inhibition by DMHCA revealed in the present work should not be viewed as a negative DMHCA effect on the retina and prevent further evaluations of DMHCA as a pharmaceutical for the treatment of retinal diseases. First, moderate accumulation of desmosterol does not seem to be toxic as indicated by apparently healthy heterozygous carriers of the DHCR24 inactivating mutations. These subjects have only moderately elevated levels of plasma desmosterol, and the levels of their serum cholesterol are normal (FitzPatrick et al., 1998; Andersson et al., 2002; Zolotushko et al., 2011). Second, DHCR24 inhibition may lead to partial inhibition of cellular cholesterol biosynthesis and thus decrease intracellular cholesterol independent of the upregulation of reverse cholesterol transport genes. Third, desmosterol is an LXR agonist, and accumulation of desmosterol in macrophage foam cells was shown to activate LXRs and suppresses inflammatory gene expression, while upregulating the expression of reverse cholesterol transport genes (Spann et al., 2012). A similar mechanism could be operative in some retinal cells, e.g., microglia/macrophages. However, because of a small number of these cells in the retina, which is composed of multiple cell types (Fliesler and Bretillon, 2010), this mechanism was not detected in the present work when total mRNA was isolated from the whole retina. Finally, beneficial effects of GW3965, a first generation LXR agonist, on early stages diabetic retinopathy were already established in a mouse model (Hazra et al., 2012). Therefore, evaluations of other LXR agonists, which do not induce hepatic lipogenesis and have unique pharmacologic profile, are required. DMHCA should be tested for the effects on the retina either at higher doses or using eye-specific routes of delivery (e.g., ophthalmic eye drops or intravitreal injections). A caveat to studies leading to higher retinal DMHCA concentrations is that they may increase retinal *Dhcr24* expression via SREBP2 (Zerenturk et al., 2012), a sensor for intracellular cholesterol levels and a transcriptional activator of cellular cholesterol biosynthesis (Horton et al., 2002; Brown and Goldstein, 2009). Indeed, a decrease in retinal cholesterol in DMHCA-treated mice due to DHCR24 inhibition by DMHCA can potentially upregulate SREBP2 processing and thereby increase *Dhcr24* expression. This increase may ultimately overcome DMHCA inhibition of DHCR24 and diminish retinal cholesterol lowering.

An interesting finding of the present work is an apparent lack of DMHCA effect on the levels of retinal cholesterol esters (**Figure 6G**), which may be generated when cholesterol is in excess in a cell and are rapidly hydrolyzed (**Figure 1**) when there is a decrease in the levels of cellular cholesterol (Chang et al., 2006). ACAT1, a cholesterol esterifying enzyme present in the retina (Saadane et al., 2016), was shown to be activated by cholesterol and oxysterols on the enzyme allosteric site (Zhang et al., 2003; Liu et al., 2005). Thus, it is plausible that DMHCA, a steroidal compound, could activate ACAT1 as well. Furthermore, the retina was also shown to express lysosomal acid lipase (Elner, 2002), one of the several enzymes with cholesterol ester hydrolyzing activities (Ghosh, 2012). Accordingly, unchanged cholesterol ester levels in DMHCA-treated mice could either be due to ACAT1 activation by DMHCA or DMHCA inhibition of a cholesterol ester hydrolase. The identification of the mechanism beyond the unchanged cholesterol ester levels in the retina of DMHCA-treated mice was beyond the scope of the present work. However, we may investigate this finding in the future because it may point us to an important enzyme that controls the levels of cholesterol esters in drusen, a hallmark of AMD.

In summary, the present work identified DMHCA dosages, formulations, and routes of delivery which reduce total retinal cholesterol without increasing serum triglyceride and cholesterol levels. Notably, a reduction in total retinal cholesterol was due to a reduction in unesterified cholesterol rather than esterified cholesterol. We also show that in *Cyp27a1^−/−^Cyp46a1^−/−^* mice, a genotype with a significantly increased retinal cholesterol, DMHCA administration altered retinal levels of cholesterol precursors lanosterol, zymosterol, desmosterol, and lathosterol. This result suggests a partial inhibition of retinal DHCR24. Retinal expression of the LXR target genes was affected only moderately in DMHCA-treated mice, thus providing evidence that at the doses tested and genotypes studied, DMHCA acts in the retina mainly as an enzyme inhibitor of cholesterol biosynthesis rather than a LXR agonist.

## Author Contributions

NE-D, NM, and IP participated in research design. NE-D, AA, NM, AS, and ML conducted the experiments. NE-D, AA, NM, AS, ML, and IP performed the data analysis. IP wrote the manuscript.

## Conflict of Interest Statement

The authors declare that the research was conducted in the absence of any commercial or financial relationships that could be construed as a potential conflict of interest.

## Abbreviations

ACAT1 and ACAT2: acyl-coenzyme A:cholesterol acyltransferases 1 and 2, respectively
BW: body weight
CYP: cytochrome P450
DHCR24: Δ24-dehydrocholesterol reductase
DMHCA: *N*,*N*-dimethyl-3β-hydroxycholenamide
HPCD: 2-hydroxypropyl-β-cyclodextrin
LXR: liver X receptor
qRT-PCR: quantitative real time PCR
